# Hope During COVID-19 Lockdown

**DOI:** 10.7759/cureus.15097

**Published:** 2021-05-18

**Authors:** Dorit Redlich Amirav, Omri Besor, Israel Amirav

**Affiliations:** 1 Rehabilitation Medicine, University of Alberta, Edmonton, CAN; 2 Occupational Therapy, Tel Aviv university, Tel Aviv, ISR; 3 Pediatrics, Ichilov Hospital, Tel Aviv, ISR

**Keywords:** mental wellbeing, positive psychology, covid-19 pandemic, covid 19 impact of lockdown, general well being

## Abstract

Introduction

The COVID-19 pandemic has changed daily life in unexpected ways including strict lockdowns periods that may shape hope.

Method

This study compared hope levels among an online sample of 584 adults in late April 2020 during the COVID-19 lockdown (2020 survey) to 884 adult internet users who participated in the 2019 Hope Barometer survey which was performed six months prior to the COVID-19 pandemic (2019 survey). Both surveys used identical validated hope and depression measures.

Results

Despite high degrees of depression, hope levels slightly but significantly increased in the 2020 survey compared to the 2019 survey. Differences between the 2019 and 2020 surveys appeared across all demographic groups, with no differences related to age, sex, or education.

Conclusions

Despite the increased depression, the COVID-19 lockdown was associated with significantly higher hope levels.

## Introduction

Strict public health measures were taken to contain the global spread of the COVID-19 virus which was declared a pandemic by the WHO on March 11, 2020. The constantly increasing numbers of cases and deaths have led authorities to impose social distancing and home confinement [1]. Many employees were asked to work remotely, public places, such as restaurants, stores, and playgrounds, were closed, and schools and universities moved to remote learning. Unemployment levels soared dramatically [2], and many people experienced a myriad of difficulties, including economic stressors, social isolation, and significant mental issues [3]. In particular, increased levels of fear, anxiety, and uncertainty were frequently reported [4,5].

Hope has been increasingly recognized as an important positive factor in wellbeing, and it has been suggested to play a major role in times of uncertainty [6]. Hope has been shown to be an important resource in coping with stress and uncertainties [7] and in psychological adjustment [8,9]. Hope is particularly important given the numerous uncertainties and unpredictability that characterize pandemics and lockdowns. While exponentially increasing numbers of reports of negative psychological impacts have been described [10], very little has been published on hope.

Edey and Jevne [6] defined hope as “enabling people to envision a future in which they are willing to participate” [p. 50]. COVID-19 lockdown is a dramatic example of a state in which the future has become uncertain for so many people worldwide, propelling the subject into one that warrants exploration and study. While feelings of hopelessness, despair, and fear have been extensively explored during COVID-19, no study has specifically looked into hope during the COVID-19 lockdown. Moreover, most available studies on the negative influences of the pandemic lack comparisons of these outcomes to pre-pandemic times, calling for research in order to inform health officials and public policymakers about how to assess the prevailing levels of hope and the interventions for enhancing them.

The aim of the current study, therefore, was to explore hope during COVID-19 lockdown by comparing two large samples that specifically surveyed hope levels in Israel before and during the first COVID-19 lockdown. The first survey was conducted in November 2019, and the second survey was conducted in April 2020, one month after the COVID-19 pandemic had been declared. Both surveys included the commonly used hope scales, the Dispositional Hope Scale (DHS) developed by Snyder et al. [11], and the Perceived Hope Scale (PHS) developed by Krafft et al. [12]. 

## Materials and methods

Participants

The participants of the two surveys were recruited via online advertisements on social media, as well as through public and well-known national news websites (Y-Net). The pre-COVID-19 survey was conducted during the month of November 2019, and the Hope Barometer (HB) was used to acquire data.

Survey characteristics

The HB is an annual cross-sectional internet survey that was developed by the Swiss Society for Future Studies in 2011 to determine the extent to which societies are focused on positive future threats, concerns, and fears as opposed to immediate ones. That group of European researchers from different disciplines reached the conclusion that Western society tends to focus upon immediate dangers, concerns, problems, and fears and less on the positive possibilities for the future. The HB has been in use annually for 10 years in several European countries, with more than 10,000 participants each year. The HB project has three main objectives: first, to encourage public discourse on hope in the participating countries, second, to empirically explore hope as perceived by individuals and groups in different contexts and cultures, and third, to contribute to the understanding of the theoretical concept of hope [13]. 

The HB includes four scales. Two for hope (DHS and PHS), one for depression and one for loneliness.

DHS

The DHS [11] is one of the most commonly used and most valued hope scales. According to Snyder’s theory of hope, people are generally goal-oriented and have the willpower (a measure of agency) to achieve their goals in various ways. People have some degree of control of their daily routines most of the time. When this control is taken away from them, people use more of their agency to explore different pathways to achieve their goals (a measure of pathways). Furthermore, they will also search for new goals to achieve. The DHS includes four items to assess agency (Cronbach alphas between 0.71 and 0.76), and four items to assess the cognitive dimension of pathways (Cronbach alphas between 0.63 and 0.80). The questions are rated on a 6-point Likert scale from 0 (strongly disagree) to 5 (strongly agree). The scale is relatively short, user-friendly, and has good psychometric properties, such as internal consistency, stability, and structural validity [14].

PHS

To complement the data that can be obtained from the DHS, the HB also includes the PHS [12] to measure hope as directly perceived by the individual. The PHS addresses hope in a broader sense (e.g., life satisfaction and happiness aspects) compared to the DHS, which focuses mainly on the cognitive dimension of hope. The PHS is a unidimensional scale that includes six items, such as “Hope outweighs anxiety in my life” and “I am hopeful with regard to my life” [12]. The questions are rated on a 6-point Likert scale from 0 (strongly disagree) to 5 (strongly agree). The PHS was shown to have good internal consistency, with Cronbach alphas between 0.87 and 0.89 (indicative of positive reliability and validity). The items of the DHS and PHS scales are outlined in Table 1. 

**Table 1:** Main Hope scales. DHS: Dispositional Hope Scale; PHS: Perceived Hope Scale.

Snyder DHS	Kraft PHS
1. I can think of many ways to get out of a jam.	1. In my life hope outweighs anxiety.
2. I energetically pursue my goals.	2. My hopes are usually fulfilled.
3. There are lots of ways around any problem.	3. I feel hopeful.
4. I can think of many ways to get the things in life that are most important to me.	4. Hope improves the quality of my life.
5. Even when others get discouraged, I know I can find a way to solve the problem	5. I am hopeful with regard to my life.
6. My past experiences have prepared me for my future.	6. Even in difficult times I am able to remain hopeful.
7. I’ve been pretty successful in life.	
8. I meet the goals that I set for myself.	

Depression

The PHQ-9 [15] is a nine-item depression scale of the patient health questionnaire. It is one of the most validated tools in mental health and can be a powerful tool to assist clinicians with diagnosing depression and monitoring treatment response. Respondents indicate the frequency of depression symptoms in the preceding 2 weeks on a 4-point scale, ranging from 0 (never) to 3 (nearly every day), for a total score ranging from 0 to 27, with higher scores indicating an increased likelihood of depression. The PHQ-9 demonstrated good internal reliability with a Cronbach’s α of 0.89 [15].

Loneliness

The loneliness scale [16] used in this study is a 5-item self-report measure from the NIH Toolbox Adult Social Relationship Scales that has excellent internal reliability with a Cronbach’s α of 0.94. Participants were asked, for example, how often in the last month “I feel alone and apart from others” and “I feel left out” [16].

Procedures

We used the same survey for the COVID-19 lockdown period that was used in November 2019. Both anonymous surveys also included the HB outcome measures of Depression and Loneliness, and they both collected demographic information on participants, including age, sex, religion, marital status, the presence of children under age 18 years in the household, education level, and occupation. The COVID-19 survey was conducted throughout the last two weeks of April 2020, during the middle of the first lockdown period in Israel. With stay-at-home directives beginning in early March, most participants had experienced life changes for more than one month at the time of the survey.

Ethics

The ethics committee of Tel Aviv University reviewed and approved this study (approval no. 0001254-1). The anonymous surveys were distributed by means of various media platforms, and the participants completed the survey after providing their informed consent.

Statistical analysis

The descriptive statistics involved absolute numbers and frequencies (%) for categorical variables. The chi-square test or Fisher’s exact test was used to assess group diﬀerences. An independent sample T-test was used (under normality assumption due to the central limit theorem) to examine differences of the continuous variables (DHS score, PHS score, Loneliness score, and Depression score) between the 2019 and 2020 groups. The Pearson correlation coefficient (r) was used as a measure of the strength of the association between these outcome measures. Data were considered statistically signiﬁcant when p < .05. Statistical analyses were performed with IBM SPSS statistics, v.25 (Chicago, IL).

## Results

A total of 1,468 individuals were included in this study, of which 884 completed the survey during November 2019 and 584 individuals completed the survey during April 2020. Their demographic data are presented in Table 2.

**Table 2:** Demographic characteristics.

Characteristic		2019	2020 (COVID-19)	p-value
N		884	584	
Age, mean (SD), years		40.74 (15.796)	39.09 (15)	0.044
Sex, N (%)	Male	262 (29.6)	186 (31.8)	0.368
	Female	622 (70.4)	398 (68.2)	
Children, N (%)	None	201 (22.7)	140 (24)	0.583
	Yes	683 (77.3)	444 (76)	
Education, N (%)	No university	173 (19.6)	117 (20)	0.827
	University	711 (80.4)	467 (80)	
Occupation, N (%)	Unemployed	249 (28.17)	166 (28.4)	0.915
	Employed	635 (71.83)	418 (71.6)	
Religion, N (%)	Religious	271 (30.7)	180 (30.8)	0.37
	Secular	610 (69)	404 (69.2)	
Marital status, N (%)	Single	200 (58.7)	141 (41.3)	0.5
	In relationship	684 (60.7)	443 (39.3)	

Apart from a minimal statistical difference in age, both groups were similar in the selected characteristics. Data on the mean outcome measures are presented in Table 3.

**Table 3:** Outcome measures. DHS: Dispositional Trait Hope Scale; PHS: Perceived Hope Scale.

Measure	2019		2020 (COVID-19)	p-value		
DHS total score, mean (SD)	29.83 (6.158)	31.38 (4.288)		<0.001		
PHS total score, mean (SD)	25.85 (6.134)	27.52 (3.637)		<0.001		
Loneliness, mean (SD)	11.06 (4.318)	10.36 (3.797)		=0.001		
Depression, mean (SD)	2.94 (2.758)	3.34 (2.408)		=0.003		

The mean DHS score was 29.83 (out of a maximum of 40) in 2019 and rose to 31.38 in 2020 (p < 0.001), indicating that hope was relatively high in both years but significantly higher during COVID-19. The mean PHS score was 25.85 (out of a maximum of 35) in 2019 and rose to 27.52 in 2020 (p < 0.001), indicating that hope was relatively high in both years but significantly higher during COVID-19. A maximum score of 25 represented a high degree of loneliness, and the mean score of loneliness was 11.06 in 2019 and it fell to 10.36 in 2020 (p = 0.001). A maximum score of 12 represented a high degree of depression, and the mean depression score was 2.94 in 2019 and it rose to 3.34 in 2020 (p = 0.003), indicating that although both 2019 and COVID-19 depression levels were relatively low, they rose significantly during COVID-19.

**Figure 1:**
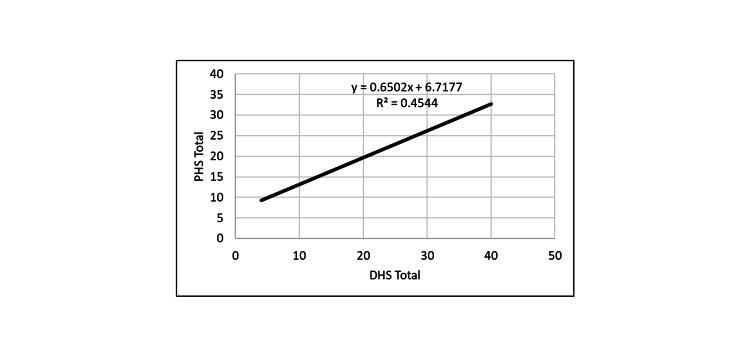
Correlation between PHS and DHS. PHS: Perceived Hope Scale; DHS: Dispositional Hope Scale.

**Figure 2:**
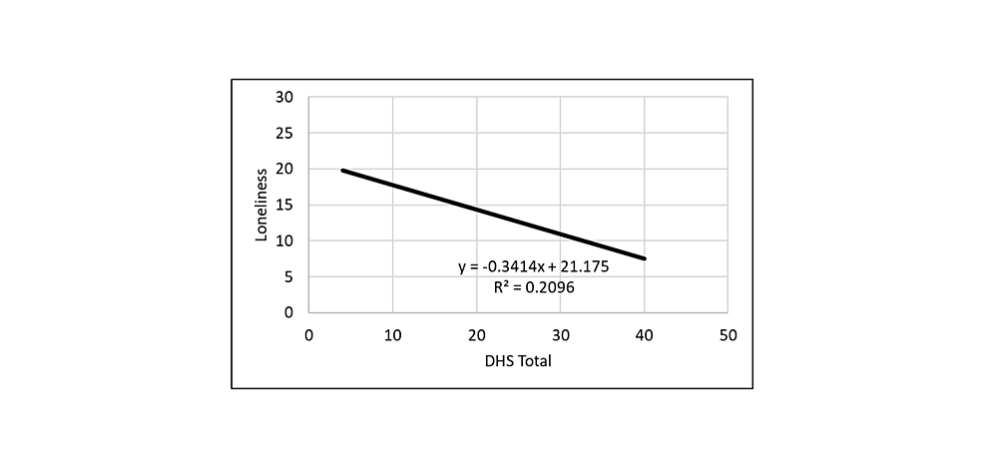
Correlation between loneliness and DHS. DHS: Dispositional Hope Scale.

**Figure 3:**
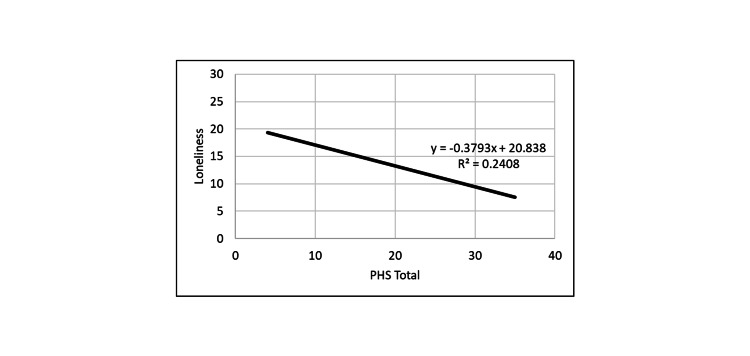
Correlation between loneliness and PHS. PHS: Perceived Hope Scale.

**Figure 4:**
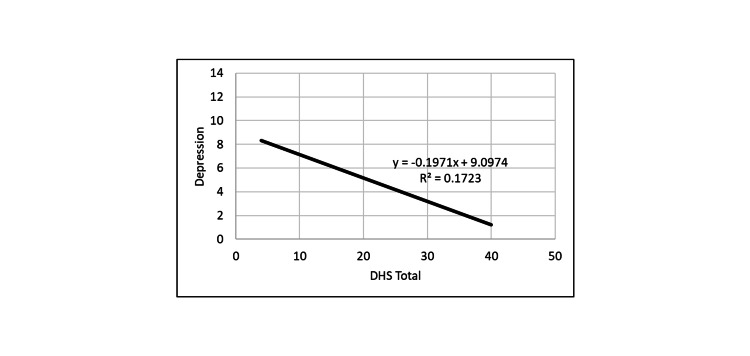
Correlation between depression and DHS. DHS: Dispositional Hope Scale.

**Figure 5:**
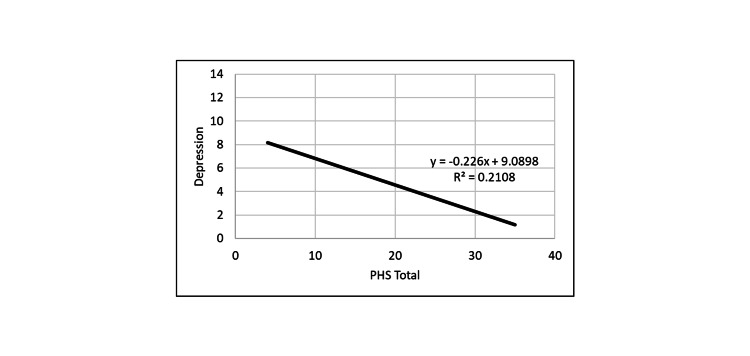
Correlation between depression and PHS. PHS: Perceived Hope Scale.

No significant correlations were found between any of the demographic data to the hope measures. However, there were significant correlations between all outcome measures (Tables 4-6 and Figures 1-5). 

**Table 4:** Linear correlations (N = 1,468). DHS: Dispositional Hope Scale; PHS: Perceived Hope Scale.

Pearson correlation p-value	DHS	PHS	Loneliness	Depression
DHS	1	0.674	-0.458	-0.415
		p < 0.001	p < 0.001	p < 0.001
PHS		1	-0.491	-0.459
			p < 0.001	p < 0.001
Loneliness			1	0.490
				p < 0.001
Depression				1

**Table 5:** Linear correlations 2019 (N = 884). DHS: Dispositional Hope Scale; PHS: Perceived Hope Scale.

Pearson correlation p-value	DHS	PHS	Loneliness	Depression
DHS	1	0.675	-0.473	-0.439
		p < 0.001	p < 0.001	p < 0.001
PHS		1	-0.477	-0.468
			p < 0.001	p < 0.001
Loneliness			1	0.515
				p < 0.001
Depression				1

**Table 6:** Linear correlations 2020 (N = 583). DHS: Dispositional Hope Scale; PHS: Perceived Hope Scale.

Pearson correlation p-value	DHS	PHS	Loneliness	Depression
DHS	1	0.645	-0.410	-0.418
		p < 0.001	p < 0.001	p < 0.001
PHS		1	-0.537	-0.534
			p < 0.001	p < 0.001
Loneliness			1	0.470
				p < 0.001
Depression				1

PHS positively correlated with DHS, and both hope measures were inversely correlated with loneliness and depression measures. Thus, the greater the hope level, the smaller was the depression and loneliness levels.

## Discussion

The results of this study showed a slight but nevertheless significant increase in hope levels of Israeli survey responders during the COVID-19 lockdown. The COVID-19 pandemic is an example of one of the most serious worldwide situations that humanity has encountered over the last century. Due to the uncertainty of the unknown tomorrow, lockdowns may have an enormous psychological impact on an unprecedented scale. People who are forced to change their day-to-day activities due to lockdown may face many difficulties in a myriad of life domains, such as psychological and physical health, financial upheavals, and more [17]. The pandemic, in general, may be associated with significant stress, anxiety, and fear centering around a new disease and its unknown consequences. People may feel overwhelmed by strong emotions. Beckman observed that the inability to continue daily routines and practice usual habits and work activities often leads to low levels of energy, chaos, and emotional exhaustion [18]. As might be expected, many of the reports in the literature emerging from the COVID-19 pandemic in general focus on negative effects, such as despair, helplessness, and hopelessness and apply various tools and questionnaires to measure these negative effects [3,19]. To the best of our knowledge, this is the first study that specifically explored the association of COVID-19 lockdown with hope.

There are various ways to measure hope [14]. In this study, we used Snyder’s and Krafft’s hope scales. As previously mentioned, Snyder’s DHS scale is based on an agency and pathways theory of hope, while Kraft’s PHS scale reflects a broader view of hope that includes emotions, life satisfaction, and happiness. To complement the information that can be obtained from the Snyder DHS scale, the Kraft PHS scale provides broader psychological aspects, which may be at least as relevant to the current situation. One would expect that hope would be reduced during such difficult times where depression, anxiety, and uncertainty about the future are on the rise. However, the results of the current study showed the opposite. None of the specific hope scales showed any reduction of hope and, although not to a great extent, both scales demonstrated significantly higher levels of hope during COVID-19 lockdown compared to pre-COVID-19 findings.

How can this be explained? Hope theorists [20,21] have long suggested that hope never vanishes but rather exists implicitly and unnoticed in our everyday life. It is only when we face a stressful and fearful situation like the COVID-19 pandemic, will we start searching for hope [22]. From that perspective, hope is metaphorically similar to the air we breathe: air is taken for granted in our daily life until we are suffocating and struggling to breathe. Similarly, COVID-19 may not only be a physical lack of air and oxygen. Being associated with stress, anxiety, uncertainty about the future and all the previously mentioned negative psychological impacts, the COVID-19 pandemic may also hint at the beginning of the hope search.

Hope is not a fixed entity but rather one that can change and being shaped by time and context [20,23]. For example, Eliott and Olver [23] found that hope was context-related and changed as situations changed in a study of the experiences of terminal cancer patients. Those authors suggested that hope is flexible, elusive, vulnerable, and changeable. Another study on parents of children with cancer showed that their hope continuously changed over an 18-month study period [24]. Dorsett [22] for instance, stated that the meaning of hope had changed over a period of 10 years among patients who coped with spinal cord injury. After first hoping for a complete recovery, they later hoped for a satisfying quality of life. Several studies suggested that hope is inherently on a spectrum ranging from hopelessness to hope [20].

In a recent study conducted during the COVID-19 pandemic, Twenge and Joiner [3] showed an increased degree of stress and hopelessness in a large sample of US adults compared to a similar survey done in 2018. At first glance, this may seem contrary to the present results, but given the nature of hope as described in the literature, this discrepancy can be explained: describing oneself as being in a hopeless state does not mean that hope has vanished. The finding that hopelessness increased between 2018 and 2020 in Twenge’s study does not rule out the possibility that hope also increased during 2020. This likelihood was supported by a study by Peterson and Seligman [25] of US adults following the terrorist attacks of September 11, 2001 (9/11). Many individuals across the country reported elevated distress after the 9/11 attacks [26], but Peterson and Seligman [25] asked specifically about the impact of positive aspects, including hope, associated with that event. Those authors found that the scores for hope significantly increased two months after this traumatic event compared with scores for those individuals who completed the survey before September 11 [25].

Although the sense of loneliness was relatively moderate in both the 2019 and COVID-19 surveys in our study, it decreased significantly during the COVID-19 lockdown. This is in line with several recent findings that showed that despite negative personal effects of home confinement during COVID-19, social cohesion and connectedness increased and loneliness decreased. These latter factors have long been closely related to goal-directed behavior and play a major role in increasing hope [9]. Although people could not meet physically and personally during the lockdown, they found creative and innovative ways for emotional connections [27]. The need to work from home created increased free and leisure time and resulted in spending more time on virtual social platforms such as WhatsApp and Zoom, reflecting new ways to “stay in touch”. Furthermore, people in Italy and Israel went out on their balconies and rooftops and clapped hands, danced, played instruments, sang, and hung banners inscribed with messages of hope and thanks to the medical professionals [28]. Imber‐Black [29] described a grandmother clapping hands with her granddaughter on a balcony in Rome saying: “It was from our hearts, to say thanks and show we can get past this…” All of these actions may not only explain why hope did not disappear but also why the perceived loneliness observed in our study participants had diminished.

Some limitations to our study need to be acknowledged, beginning with its relatively small sample size. Nevertheless, the differences in the levels of hope reached statistical significance, thus suggesting a real effect. Our findings are limited by the use of a cross-sectional study design that does not provide the causal associations among the study variables. We also used convenience sampling in order to recruit the participants, thus those who responded might have a specific interest in the topic. Another drawback is that we had no way to know how many of the responders participated in both anonymous surveys. Finally, the context and impacts of COVID-19 lockdowns may be different among countries, thus generalization of the findings may be difficult.

## Conclusions

The present study provides the first glimpse of levels of hope during the lockdown and should be replicated using a more traditional probability sample. Given that hope helps to deal with uncertainties, difficulties, and stressful situations, the results of this study suggest that it may be important to explicitly enquire into hope and to encourage societies to use a language of hope during such situations and even develop specific hope-focused interventions to mitigate the psychological impact of this and future pandemics.
